# Experience with a Wearable Cardioverter-defibrillator in 436 Patients

**DOI:** 10.19102/icrm.2022.130104

**Published:** 2022-01-15

**Authors:** Herbert Nägele, Eike Groene, Daniel Stierle, Matin Ali Khan, Matthias P. Nägele

**Affiliations:** ^1^Albertinen Cardiovascular Center, Hamburg, Germany; ^2^Department of Cardiology, University Hospital of Zürich, Rämistrasse, Switzerland

**Keywords:** Heart failure, sudden cardiac arrest, sudden cardiac death, ventricular tachycardia, wearable cardioverter-defibrillator

## Abstract

The aim of the present study was to review the safety and efficiency of wearable cardioverter-defibrillators (WCDs) under current guideline-directed medical therapy (GDMT). We retrospectively analyzed 436 consecutive WCD patients seen in the years 2014–2020. Detected automatic arrhythmia alarm (AA) episodes were validated and classified as correct or incorrect. The positive predictive value (PPV) was calculated. GDMT was optimized in our outpatient clinic to maximal tolerated doses. During a total wear time (WT) of 23,527 days, 3,135 AAs were transmitted from 206 of 436 (47.2%) patients. Visual analysis revealed correct diagnoses of non-sustained ventricular tachycardia (VT) in 38 AAs from 6 patients (total PPV, 1.21%; PPV in VT patients, 41%); the remaining AAs were artifacts. No appropriate or inappropriate shocks and fatalities were recorded. LVEF significantly improved (*P* < .001) during the WT from 25% (range, 20%–30%) to 40% (range, 34%–46%). Defibrillators were implanted in 109 patients (27%). The PPV for VT of the WCD was very low. There were fewer instances of true VT than previously reported, and no shocks (appropriate or inappropriate) were delivered. The majority of patients greatly improved with GDMT, and device implantation rates were lower than previously reported. Improvements in arrhythmia detection algorithms are warranted. Based on our results, WCDs are rarely needed for lifesaving shocks under optimal GDMT.

## Background

The idea for the external wearable cardioverter-defibrillator (WCD) system originated in the early 2000s.^1,2^ With further development of the WCD, the LifeVest^®^ WCD (Zoll Medical Corp., Chelmsford, MA, USA) was approved by the United States (US) Food and Drug Administration in 2002 and by German health authorities in 2019 (incorporated into the “Heilmittelkatalog”). It is widely and increasingly used as an alternative in patients with a high risk of sudden death, particularly when device implantation is not feasible.^3^ Some of these patients have automatic implantable cardioverter-defibrillators (AICDs) which have been explanted or deactivated due to infection or malfunction, although the majority of patients were newly diagnosed with heart failure (HF) in whom the recovery of ventricular function could be expected.^4^ According to guidelines, WCDs are indicated as a IIb recommendation in the early phase after an acute myocardial infarction and for ≥3 months after the initial diagnosis in patients with untreated HF^5,6^ or after myocarditis.^7^ Similar recommendations have been made for patients after cardiac surgery and those with a left ventricular ejection fraction (LVEF) <35%.^8^ Al-Khatib et al.^9^ stated that the right device could be selected with the help of a WCD.

In the present study, we reviewed the safety and efficiency of WCDs, as the results of the only major controlled randomized study, the Vest Prevention of Early Sudden Death Trial (VEST), showed a significant reduction in overall mortality only in an unadjusted analysis and no difference in arrhythmic deaths.^10^ Therefore, more recent information from WCD use in clinical practice is needed.

## Methods

This was a retrospective observational study conducted at a tertiary cardiology center (Albertinen Cardiovascular Center, Hamburg, Germany). The study population included 436 consecutive WCD patients seen in the years 2014–2020.

The WCD used in the present study was the LifeVest^®^ WCD, which consists of an elastic belt and shoulder straps. The electrocardiogram (ECG) is created using 4 sensors, and shock therapy is delivered by 3 electrodes, which apply the contact gel automatically immediately before the shock is delivered. The control unit is worn around the waist and contains a battery, a defibrillator, an alarm system (vibration and sound), and response buttons. In addition to being a defibrillator, the device records ECGs created during arrhythmia alarm (AA) episodes and transmits them via the Internet. At the same time, an email message is sent to the patient’s physician. Patients are able to avoid the shock delivery by pressing the response button, allowing conscious patients to prevent themselves from being inappropriately shocked in response to noise artifacts or hemodynamically stable ventricular tachycardia (VT) episodes.

Automatic AAs and daily wear times (WTs) were electronically transmitted. Any episodes detected were visually validated by two experienced cardiologists and were classified as correct or incorrect. Patients were seen in our outpatient clinic for follow-up, focusing on the optimization of guideline-directed medical therapy (GDMT), which included determining the highest tolerable doses of medications and echocardiographic LVEF measurements. Patients were also educated on the flexible usage of diuretics and lifestyle improvement through participation in our exercise rehabilitation program for HF patients. The medical therapy was documented and analyzed, comparing the kind and dosage of HF medication before the first contact (“baseline”) and at the time of WCD withdrawal (“best”). The LVEF was measured using the modified Simpson method (biplane method of disks).

After the last follow-up visit, patients were assigned to 2 different groups: patients without AICD implantation and patients with immediate AICD implantation (on the day of WCD withdrawal).

### Statistical methods

We used the WinSTAT R software, version 2012.1.0.96 (R. Fitch, Bad Krozingen, Germany) for statistical analysis and set the statistical significance at *P* values <.05. Chi-squared and Kruskal–Wallis tests were used to evaluate differences between groups, depending on the type of data. Non-normal variables were reported using median (interquartile range) values.

The present study was approved by the Hamburg Board of Ethics (registration number: WF-035/21).

## Results

**Figure 1:**
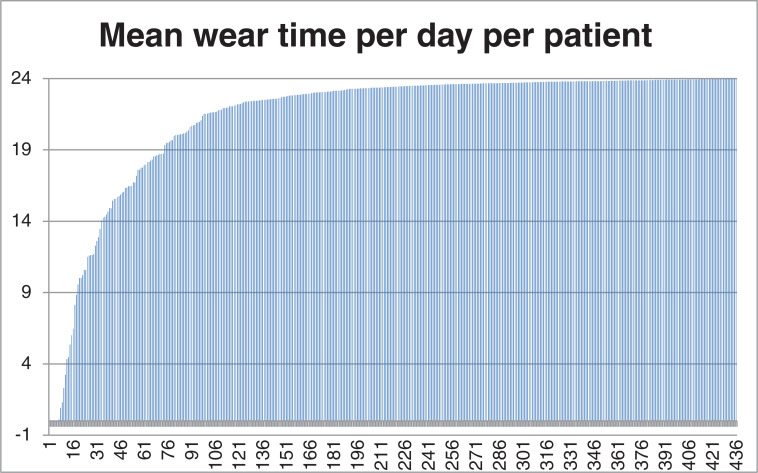
Wear time per day per patient. The x-axis shows the patient number, and the y-axis shows hours.

**Figure 2:**
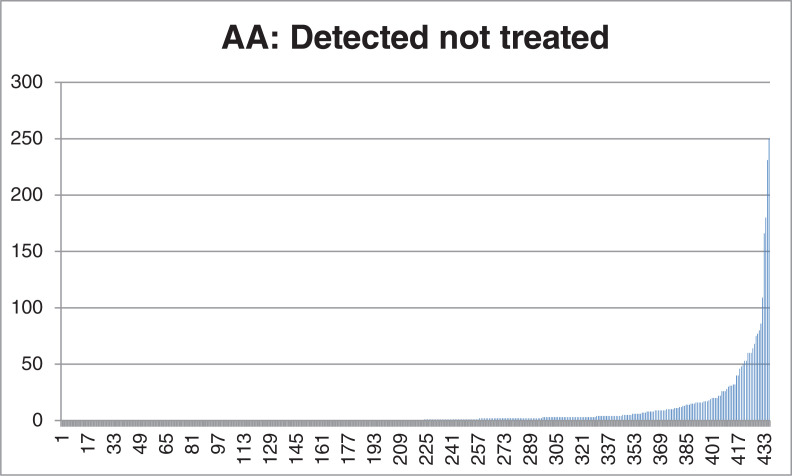
Rate of arrhythmia alarm (AA) (“detected not treated”) per patient. The x-axis shows the patient number, and the y-axis shows the number of AAs. Abbreviation: AA, arrhythmia alarm.

**Figure 3:**
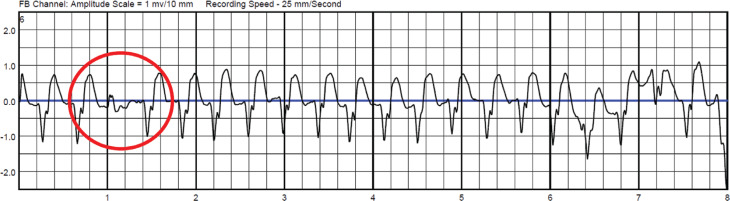
Ventricular tachycardia with a rate of 150 bpm detected by transmission of electrocardiogram. The capture beat is encircled.

For 23 of 436 patients, the indication for WCD use was infection or electrical defect of already implanted defibrillators. The remaining 413 patients received WCDs as a result of a new HF diagnosis, with 163 of 413 (39%) patients having undergone cardiac surgery. Potential reversible causes of VT were seen in six patients due to drug-induced torsades de pointes tachycardia or VT due to reversible ischemia outside an acute myocardial infarction. Patient characteristics are shown in **Table 1**. The median daily WT was 23.4 (21.8–23.8) hours, and 82% of patients had a daily WT of <20 hours **(Figure 1)**. During the median WT of 52 (34–76) days per patient, based on a total of 23,527 days, 3,135 AAs were transmitted from 206 of 436 patients (47%). Most of the episodes were concentrated in 5% of the patients **(Figure 2)**. The visual analysis performed by two experienced cardiologists revealed a correct diagnosis of non-sustained VT in 38 AAs from 6 patients (total PPV, 1.21%; PPV in VT patients, 41%); the remaining AAs were confirmed as artifacts. Changes in morphology, sudden onset, and rate stability were the criteria used to confirm VT. An example of VT with a typical capture beat is shown in **Figure 3**. All episodes of VT were hemodynamically stable, oligosymptomatic, and had a relatively low heart rate of 160 ± 9.9 bpm. The mean duration of all VT episodes was 12 ± 9 seconds. No appropriate or inappropriate shocks were delivered. The mortality rate was 0% during the observation period. For statistical analyses, 14 patients with device infection/malfunction and 38 patients who did not complete the entire follow-up were excluded, even though telemedical data were received from all patients. We optimized the GDMT in all cases.

By the end of the observation period, β-blocker therapy had increased from baseline (11%) to 98%, renin–angiotensin–aldosterone inhibitor (RAASI) administration (40% sacubitril/valsartan) from baseline (12%) to 99%, and mineral receptor antagonists (MRAs) from baseline (3%) to 90%. Loop diuretics were administered to 7% of patients at the onset of the study and to 69% at the conclusion of the study. The final mean daily dosages were as follows: metoprolol, 115 ± 58 mg (n = 316); carvedilol, 38 ± 15 mg (n = 35); bisoprolol, 6 ± 3 mg (n =39); nebivolol, 6 ± 3 mg (n = 7); ramipril, 6.5 ± 7 mg (n = 158); candesartan, 15 ± 10 mg (n = 39); sacubitril/valsartan, 216 ± 110 mg (n = 154); spironolactone, 30 ± 7 mg (n = 218); and eplerenone, 30 ± 7 mg (n = 121). Data for lisinopril, enalapril, telmisartan, valsartan, and losartan are not shown due to the low number of patients. In 384 patients with at least one in-house follow-up, LVEF significantly (*P* < .001) improved from 25% (range, 20%–30%) to 40% (range, 34%–46%) during WT. AICDs were recommended in 109 patients (27%), although 3 refused. **Table 2** shows patient characteristics, risk factors, and type and etiology of HF in the group with no indication for an AICD versus that with an indication for an AICD. **Table 3** shows the interventions and physiologic parameters in patients with no indication for an AICD (n = 279) versus those with an indication for an AICD (n = 105). In the analysis shown in **Tables 2 and 3**, 14 patients with device infection/malfunction and 38 patients who did not complete the entire follow-up were excluded; therefore, a total of 384 patients were analyzed. Patients with >1 AA episode showed a significantly longer total WT of 45 (29–68) versus 60 (44–91) days (*P* < .001).

## Discussion and conclusions

The major findings of our study were fewer instances of true VT than previously reported and that no lifesaving shocks had to be delivered. The majority of our patients greatly improved with GDMT, and device implantation rates were lower than previously reported, despite similar baseline patient characteristics, including age, LVEF, etiology of HF, and WT compliance.^10^ A total of 38 confirmed episodes of VT were found in 6 patients, all of whom subsequently received an AICD. All VT episodes were slow, non-sustained, and hemodynamically stable. The data from the present study regarding the detection of low numbers of hemodynamically stable VT episodes and a complete lack of any shock delivery contrast with the results of all other studies involving WCDs, which indicate increased rates of fast, hemodynamically unstable VT episodes and “lifesaving” shocks. A nationwide analysis performed in 2010^11^ of 3,569 patients in the US with WCDs indicated an appropriate shock rate of 1.7%. In a meta-analysis performed by Masri et al.^12^ of 28 studies including 32,426 patients, the rate of appropriate WCD therapy was 5% over three months. All of the relevant studies published from 2003–2018 had data regarding confirmed WCD interventions, although the rate of patients requiring AICDs was scarcely reported. A recent registry study from Switzerland^13^ including 456 patients reported an appropriate shock rate of 3.7% and an AICD rate of 46.5%. Recently, a study by Heimeshoff et al.,^14^ which included 100 patients who had undergone cardiac surgery, found VT episodes at a rate of 10% and lifesaving shocks at a rate of 3%. None of the patients experienced any ventricular arrhythmia after surgery. In a case series study by Skowasch et al.^15^ including 46 cardiac sarcoidosis patients wearing the LifeVest^®^ WCD, 10 patients (22%) had VT/ventricular fibrillation with shock deliveries. On the contrary, the 5 cardiac sarcoidosis patients included in the present study had neither VT episodes nor any indication for an AICD during LifeVest^®^ monitoring.

The data from the present study are in line with the overall reduction in sudden death rates in HF patients in recent years.^16^ This should lead to a more critical decision-making process for AICD placement, especially in non-ischemic patients.^17^ Arrhythmia protection in newly diagnosed or worsened HF may be less important than previously thought. The idea that protection by shock in the early phase of HF is not as important as once thought is further supported by data from the Sudden Cardiac Death in Heart Failure Trial (SCD-HeFT), in which survival curves comparing AICDs and medical therapy diverged only after 18 months.^18^

The unexpected results in the present study may be due to the vigorous optimization of GDMT in our cohort. At the end of the observation period, β-blockers and RAASI prescriptions (including sacubitril/valsartan prescriptions beginning in 2017) increased to a rate of nearly 100% and MRA prescriptions increased to 90%. In a surprising contrast, many WCD studies were performed with very poor or undefined medical therapy. For example, in the WEARIT I trial,^19^ only 27% of patients were taking a β-adrenergic antagonist, 34% were on anti-arrhythmic medications, and 45% were taking inotropes. In the Prospective Registry of Patients Using the Wearable Cardioverter Defibrillator (WEARIT II)^20,21^ involving 2,000 patients, only 24% received aldosterone receptor blockers, 70% received angiotensin-converting enzyme inhibitors or angiotensin-converting receptor 1 antagonists, 85% received β-blockers, and 42% received an AICD. In the most recent VEST trial,^10^ medical therapy was not mentioned at all.

Therefore, the authors believe that patients included in those studies were not adequately treated with GDMT and should not represent the patients who are currently being prescribed a WCD. We observed an improvement in LVEF under our GDMT, crossing the “magical border” of 35% in 73% of cases, where 35% of patients showing an improvement of more than 45% can be defined as having “recovered” LVEF.^22^ In particular, patients with tachymyopathy improved and none of them required a defibrillator. Only 27% of patients had an indication for AICD implantation in the present study. Patients with an indication for AICD placement had more severe pre-existing HF, and, not surprisingly, their LVEF improved significantly less. Lower baseline heart rate and blood pressure, together with similar LVEF in patients with a subsequent AICD implantation, suggest that the capacity for improvement is partly decreased compared to the group with no indication for an AICD. Additionally, the significantly broader baseline QRS widths in patients who subsequently received AICDs may reflect a more advanced structural heart damage. In our opinion, the role of the WCD may evolve more toward that of a long-term monitoring system and reminder for compliance with a comprehensive HF program.^23^ Similarly, a follow-up study of the VEST trial showed that patients with better compliance benefit more from WCD use.^24^ Therefore, perhaps WT compliance translates to improved medical and behavioral compliance.^25^ It is possible that patients who wear WCDs take their medications correctly in an effort to graduate from the vest as quickly as possible.

Furthermore, the PPV for VT of the WCD was found to be very low. AA artifacts, mostly related to the motion of the leads, are a major problem in the reliable detection of arrhythmias, and current integrated noise reduction algorithms seem to be insufficient. Inappropriate repetitive alarms become a psychological problem, requiring patients to press the STOP button to suppress painful shocks. Therefore, a high number of false AAs were transmitted, resulting in a low PPV for VT, which was too low for accurate usage. As artifacts were concentrated only in certain patients, individual factors of device usage may be the mechanism causing the artifacts. A comparison of patients with ≤1 AA episode to those with ;1 episode showed that, not surprisingly, the total WT is strongly related to such artifacts. Perhaps, in the long run, the WCD becomes inconvenient, and patients try to overcome this by making changes, which then lead to artifacts.

In summary, the majority of the WCD patients in the present study greatly improved under GDMT, and permanent device implantation was avoided. In our opinion, WCDs have to be accompanied by a comprehensive HF management program. In this regard, new features of the WCD are currently under development which may be of interest, such as the ability to detect heart rate changes, rales, thoracic impedance, activity logs, and body position measurements. These possible features carry the potential to steer the WCD away from being simply a “shock machine” to a well-rounded HF treatment tool. Furthermore, improvements in arrhythmia detection algorithms are needed.

### Limitations

The results of the present study stem from a single center; therefore, selection bias of patients may have been a problem. Another possible reason for the low rate of AA episodes in the present study is patient selection—a lot of patients selected for the WCT most likely had a low risk of sudden cardiac death in general. Nearly half of the patients enrolled in this series would be expected to have very low rates of VT due to having valvular heart disease, DCM, and tachycardia-mediated cardiomyopathy. Of the study population, 38 patients (8.7%) were excluded due to a lack of follow-up with regard to their LVEF workup; however, their alarm transmissions were received and included in the analysis. Our study covers a limited time period, and, therefore, a study involving a long-term follow-up of former WCD wearers would be beneficial.

## Figures and Tables

**Table 1:** Patients’ Characteristics at Baseline (N = 436)

Age (years)	64 ± 12
Men (%)	81
Median wear time (days)	52 (34–76)
Mean wear time per day (h)	23.4 (21.8–23.8)
Risk factors	
Body mass index (kg/m^2^)	26.5 (23–30)
Hypertension (%)	53
Diabetes (%)	23
Hyperlipidemia (%)	27
Smoking (%)	40
Alcoholism (%)	9
Indication type (%)	
Worsening of existing heart failure	14
Newly diagnosed heart failure	65
Acute coronary syndrome	14.7
Potential reversible cause of VT	1
Device infection/defect	5.3
Underlying disease (%)	
DCM	27.5
CAD	48
VCM	11.7
TCM	9.6
Acute coronary syndrome	14.4
Sarcoidosis	1.1
Myocarditis	1.1
Prior heart surgery (%)	
Heart surgery	39
CABG	15.1
CABG + MVR	3.4
CABG + AVR	3.7
AVR	6.2
TAVI	3.7
MVR	2.3
MitraClip	1.4

*Abbreviations:* AVR, aortic valve replacement; CABG, coronary artery bypass grafting; CAD, coronary artery disease; DCM, dilated cardiomyopathy; MVR, mitral valve reconstruction; TAVI, transfemoral aortic valve replacement; TCM, tachymyopathy; VCM, valvular cardiomyopathy; VT, ventricular tachycardia.

**Table 2:** Patients’ Characteristics, Risk Factors, and Type and Etiology of Heart Failure

Parameter	No AICD Implantation (n = 279)	AICD Implantation (n = 105)	*P* value
Age (years)	63.8 ± 12.4	64.7 ± 12	ns
Body mass index (kg/m^2^)	26.7 (23.7–30.5)	26.4 (23.8–29.8)	ns
Serum creatinine (mg/dL)	1 (0.9–1.2)	1 (0.9–1.3)	ns
Wear time (days)	49 (33–66)	1,558 ± 1,100	ns
VT (n)	0	6	< .001
Risk factors (%)			
Men	82	78	ns
Hypertension	52	56	ns
Diabetes	23	22	ns
Hyperlipidemia	25	33	ns
Smoking	40	36	ns
Alcohol consumption	10	6	ns
Indication for WCD (%)			
Worsening of existing HF	13.5	20	.02
Newly diagnosed HF	72.8	60	.02
Acute coronary syndrome	13	18	ns
Potential reversible cause of VT	0.7	3	<.01
Etiology of HF (%)			
DCM	27	32	ns
CAD	36	46	<.01
VCM	11.7	10.9	ns
TCM	15	0	<.0001
Sarcoidosis	1.1	1.0	ns
Myocarditis	1.5	1.0	ns
Miscellaneous	7.7	9.1	ns

*Abbreviations:* AICD, automatic implantable cardioverter-defibrillators; CAD, coronary artery disease; DCM, dilated cardiomyopathy; HF, heart failure; ns, non-significant; TCM, tachymyopathy; VCM, valvular cardiomyopathy; VT, ventricular tachycardia.

**Table 3:** Interventions and Physiologic Parameters in Patients According to Automatic Implantable Cardioverter-defibrillator Status

Parameter	No AICD Implantation (n = 279)		AICD Implantation (n = 105)		*P* value
Surgical/medical					
Heart surgery (%)*	40		35		ns
β-blocker baseline (%)	17.8		9.8		ns
β-blocker best (%)	97.4		99		ns
RAASI baseline (%)	10.2		15.8		ns
ACE best (%)	46	RAASI + ARNI 99	36	RAASI + ARNI 96	ns
AT1A best (%)	15		17		ns
ARNI best (%)	38		43		ns
ARB baseline (%)	1.8		5.9		ns
ARB best (%)	90.1		91.1		ns
Physiologic parameters					
HR baseline (bpm)	97 (80–119)		90 (76–109)		<.01
HR best (bpm)	70 (63–79)		71 (65–80)		ns
SBP baseline (mmHg)	127 (114–140)		120 (107–138)		<.01
SBP best (mmHg)	120 (110–140)		120 (107–131)		ns
DBP baseline (mmHg)	80 (70–90)		74 (66–85)		<.01
DBP best (mmHg)	80 (70–80)		75 (66–80)		<.01
AF baseline (%)	42		34		ns
AF best	17		15		ns
QRS width baseline (ms)	106 (93–131)		120 (100–153)		<.01
QRS width best (ms)	100 (90–130)		120 (100–153)		<.01
LVEF baseline	25 (20–30)		22 (20–29)		ns
LVEF best	45 (40–50)		28 (24–32)		<.001
Serum creatinine (mg/dL)	1 (0.9–1.2)		1 (0.9–1.3)		ns

*Abbreviations:* ACE, angiotensin angiotensin-converting enzyme inhibitors; AF, atrial fibrillation; AICD, automatic implantable cardioverter-defibrillators; ARB, aldosterone receptor blocker; ARNI, angiotensin receptor neprilysin inhibitor; AT1A, angiotensin-converting receptor 1 antagonists; bpm, beats per minute; DBP, diastolic blood pressure; HR, heart rate; LVEF, left ventricular ejection fraction; RAASI, renin–angiotensin–aldosterone inhibitors; SBP, systolic blood pressure; VT, ventricular tachycardia.

*Among patients undergoing heart surgery, the indication for a wearable cardioverter-defibrillator was made prior to the discharge of a cardiac surgical intervention.
